# Hip Fracture in People with Erectile Dysfunction: A Nationwide Population-Based Cohort Study

**DOI:** 10.1371/journal.pone.0153467

**Published:** 2016-04-14

**Authors:** Chieh-Hsin Wu, Yi-Ching Tung, Tzu-Kang Lin, Chee-Yin Chai, Yu-Feng Su, Tai-Hsin Tsai, Cheng-Yu Tsai, Ying-Yi Lu, Chih-Lung Lin

**Affiliations:** 1 Graduate Institute of Medicine, College of Medicine, Kaohsiung Medical University, Kaohsiung, Taiwan; 2 Department of Neurosurgery, Kaohsiung Medical University Hospital, Kaohsiung Medical University, Kaohsiung, Taiwan; 3 Department of Public Health and Environmental Medicine, College of Medicine, Kaohsiung Medical University, Kaohsiung, Taiwan; 4 Department of Neurosurgery, Chang Gung Memorial Hospital at Linkou, Chang Gung University, Kweishan, Taoyuan, Taiwan; 5 Department of Pathology, Kaohsiung Medical University Hospital, Kaohsiung Medical University, Kaohsiung, Taiwan; 6 Department of Pathology, Faculty of Medicine, College of Medicine, Kaohsiung Medical University, Kaohsiung, Taiwan; 7 Institute of Biomedical Sciences, National Sun Yat-Sen University, Kaohsiung, Taiwan; 8 Department of Dermatology, Kaohsiung Veterans General Hospital, Kaohsiung, Taiwan; 9 Cosmetic applications and management department, Yuh-Ing Junior College of Health Care & Management, Kaohsiung, Taiwan; 10 Department of Neurosurgery, Faculty of Medicine, College of Medicine, Kaohsiung Medical University, Kaohsiung, Taiwan; Medical University Innsbruck, AUSTRIA

## Abstract

The aims of this study were to investigate the risk of hip fracture and contributing factors in patients with erectile dysfunction(ED). This population-based study was performed using the Taiwan National Health Insurance Research Database. The analysis included4636 patients aged ≥ 40 years who had been diagnosed with ED (International Classification of Diseases, Ninth Revision, Clinical Modification codes 302.72, 607.84) during 1996–2010. The control group included 18,544 randomly selected age-matched patients without ED (1:4 ratio). The association between ED and hip fracture risk was estimated using a Cox proportional hazard regression model. During the follow-up period, 59 (1.27%) patients in the ED group and 140 (0.75%) patients in the non-ED group developed hip fracture. After adjusting for covariates, the overall incidence of hip fracture was 3.74-times higher in the ED group than in the non-ED group (2.03 vs. 0.50 per 1000 person-years, respectively). The difference in the overall incidence of hip fracture was largest during the 3-year follow-up period (hazard ratio = 7.85; 95% confidence interval = 2.94–20.96; *P* <0.0001). To the best of our knowledge, this nationwide population-based study is the first to investigate the relationship between ED and subsequent hip fracture in an Asian population. The results showed that ED patients had a higher risk of developing hip fracture. Patients with ED, particularly those aged 40–59 years, should undergo bone mineral density examinations as early as possible and should take measures to reduce the risk of falls.

## Introduction

Erectile dysfunction (ED), a common sexual problem in men, is defined as the inability to achieve or sustain a penile erection for satisfactory sexual performance. Erectile dysfunction is a highly prevalent global health problem that considerably impacts quality of life in middle-aged men. Approximately 50% of all men older than 40 years experience some degree of ED [1]. By 2025, an estimated 322 million men are expected to suffer from ED [2]. The most common cause of ED is arterial occlusion of atherosclerosis, which also affects the coronary arteries and can potentially lead to myocardial infarction (MI) and vascular events such as stroke and peripheral arterial disease [2–7].

Hip fracture is a withering event in which subsequent functional disability and morbidity can contribute to high medical expenditures, severe health problems, and even mortality [8]. Cooper et al. estimated that 1.66 million instances of hip fractures occurred in 1990, and this number is expected to increase to 6.26 million by 2050 [9]. Hip fractures are considered the most severe osteoporotic fractures. They increase morbidity rates and negatively affect quality of life. In elderly people, the main causes of hip fracture are osteoporosis and low-impact trauma such as falls. The likelihood of a hip fracture depends on the strength of the bone and on the amount of stress sustained by the bone in a fall [10]. Because there are common pathophysiological mechanisms of the bone and vasculature and because the calcification process in vascular walls is similar to the bone formation process [11,12], coronary heart disease (CHD) is a noted risk factor for hip fracture. Since both ED and hip fracture are also associated with CHD risk factors, we hypothesized that ED is associated with hip fracture. Therefore, a nationwide population-based cohort in Taiwan was used to investigate the risk of hip fracture in patients with ED.

## Methods

### Database

This population-based cohort study used data which were obtained from Taiwan’s National Health Insurance Research Database (NHIRD), which has been described in detail previously [13–16]. The NHIRD contains administrative and health claims data collected through the National Health Insurance (NHI) programme and provides researchers with relevant claims information for each patient, including gender, registry of medical services, prescriptions, and date of birth. This study analyzed one subset of the NHIRD, the Longitudinal Health Insurance Database 2010, which comprises 1996–2010 data for 1,000,000 beneficiaries randomly sampled from the original NHIRD. The large size of the database afforded a unique opportunity to study osteoporosis risk in ED patients. In this study, diseases were classified using the diagnostic codes of the International Classification of Diseases, Ninth Revision, Clinical Modification (ICD-9-CM).

### Ethical approval

The study was performed in accordance with the Declaration of Helsinki. The study design was also evaluated and approved by the Institutional Review Board of Kaohsiung Medical University Hospital [KMUHIRB-EXEMPT (I)-20150039].

### Study population

The study cohort comprised 4636 male patients aged ≥ 40 years who had been diagnosed with psychogenic ED (ICD-9-CM 302.72) or organic ED (ICD-9-CM 607.84) during 1996–2010. Similar inclusion methods for ED have been used in relevant studies and are considered valid [13–16]. To ensure high accuracy of data, the analysis was limited to patients who had received ≥ 2 ED diagnoses during ambulatory visits or ≥1 ED diagnoses during inpatient care; additionally, only cases in which the ICD-9codes had been assigned by an urologist were included. The index date was the date of the first clinical visit in which ED was diagnosed. The exclusion criteria were history of hip fracture (ICD-9-CM 820) before the index date, female gender, incomplete information, and age younger than 40 years. To enhance the power of statistical tests and to ensure that the number of hip fracture cases was sufficient for stratified analyses, the non-ED group comprised four additional NHI beneficiaries without ED, who were randomly selected and frequency-matched by age and index year (i.e., the year of ED diagnosis) to each patient with ED. Thus, the non-ED cohort comprised 18,544 patients.

### Outcome and comorbidities

Patients in both the ED and non-ED cohorts were followed up until a diagnosis of hip fracture, censored due to loss to follow up, withdrawal from insurance, death, or the end of 2010 (i.e., end of the study period), whichever occurred first. Comorbidities identified by claims records data at baseline (before the index date) included hypertension (ICD-9-CM 401–405), diabetes mellitus (ICD-9-CM 250), hyperlipidaemia (ICD-9-CM 272), chronic kidney disease (ICD-9-CM 582, 583, 585, 586, and 588), chronic liver disease (ICD-9-CM 456, 571, and 572), chronic pulmonary disease (ICD-9-CM 490–496), hyperthyroidism (ICD-9-CM 242), hyperparathyroidism (ICD-9-CM 252), osteoporosis (ICD-9-CM 733), depression (ICD-9-CM 296.2, 296.3, 300.4, and 311), stroke (ICD-9-CM 430–438), epilepsy (ICD-9-CM 345), dementia (ICD-9-CM 290, 294.1, 331.0, and 331.2), Parkinson’s disease (ICD-9-CM 332), wrist fracture (ICD-9-CM 813, 814, 818, and 819), vertebral fracture (ICD-9-CM 805–806), and rib fracture (ICD-9-CM 807). Charlson comorbidity index (CCI) scores were used to assess the severity of the following comorbidities: MI; congestive heart failure; peripheral vascular disease; cerebrovascular disease; dementia; chronic pulmonary disease; rheumatic disease; peptic ulcer disease; mild, moderate, or severe liver disease; diabetes with and without chronic complication; hemiplegia or paraplegia; renal disease; malignancies other than skin malignancies, including lymphoma and leukaemia; metastatic solid tumours; human immunodeficiency virus infection and acquired immune deficiency syndrome. The CCI scores were then categorised into four levels: 0, 1–2, 3–4, and ≥ 5.The analysis also included the use of oral corticosteroids or testosterone.

### Statistical analysis

The distributions of categorical demographics and clinical characteristics were compared between the ED and non-ED cohorts by chi-square test. Student *t* test and Wilcoxon rank-sum test were used to compare mean age and follow-up time (y) between the cohorts, as appropriately. Cumulative incidence rates were estimated by Kaplan–Meier method, and the differences between the curves were tested by two-tailed log-rank test. Incidence rates of hip fracture per 1000 person-years were compared between the cohorts. Univariate and multivariate Cox proportional hazard regression models were used to investigate the hazard ratios (HRs) and 95% confidence intervals (CIs) for hip fracture. The multivariate Cox models were adjusted for age, CCI, and relevant comorbidities. In two-tailed tests, *P* < 0.05 was considered statistically significant. All data processing and statistical analyses were performed using Statistical Analysis Software version 9.4 (SAS Institute, Cary, NC, USA).

## Results

### Baseline characteristics of ED and non-ED cohorts

**Fig 1:**
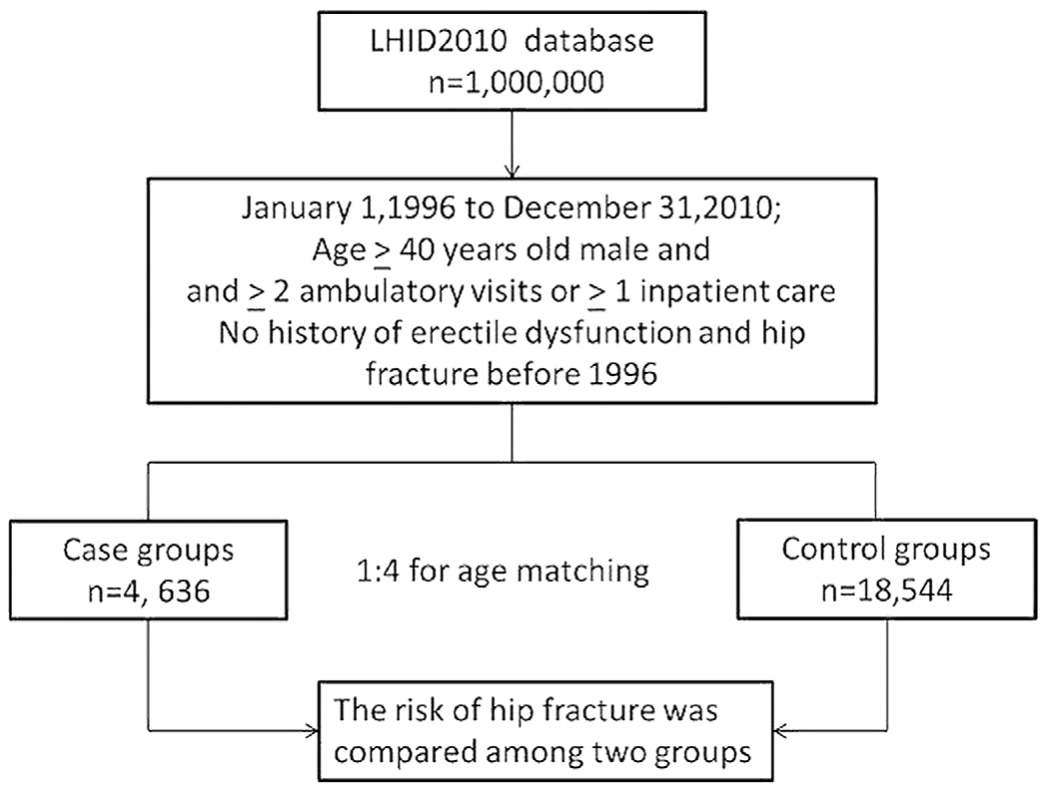
Flow diagram of the present study from the National Health Insurance Research Database in Taiwan. LHID = Longitudinal Health Insurance Database.

Fig 1 shows that the 23,180 patients enrolled in this study included 18,544 patients in the non-ED (control) cohort and 4636 patients in the ED (case) cohort.

Table 1 summarises the baseline demographic characteristics and comorbidity status of patients in ED and non-ED cohorts. The ED and non-ED cohorts had a similar mean age (58.0 ± 10.8 [mean ± SD] years and 58.0 ± 10.4 years, respectively). The largest age group was 50–59 years (33.67%) followed by 40–49 years (25.84%). The following comorbidities were significantly more likely in the ED cohort versus the non-ED cohort, respectively: hypertension (67.56% vs. 44.61%, *P* < 0.0001), diabetes mellitus (41.48% vs. 21.77%, *P* < 0.0001), hyperlipidaemia (61.95% vs. 37.28%, *P* < 0.0001), osteoporosis (10.09% vs. 3.67%, *P* < 0.0001), chronic kidney disease (23.06% vs. 10.34%, *P* < 0.0001), chronic liver disease (54.85% vs. 35.01%, *P* < 0.0001), chronic pulmonary disease (58.02% vs. 36.86%, *P* < 0.0001), hyperthyroidism (5.16% vs. 2.31%, *P* < 0.0001), dementia (7.18% vs. 2.23%, *P* < 0.0001), stroke (14.69% vs. 5.18%, *P* < 0.0001), epilepsy (3.24% vs. 1.89%, *P* < 0.0001), depression (20.56% vs. 7.77%, *P* < 0.0001), Parkinson’s disease (4.21% vs. 1.51%, *P* < 0.0001), and CCI (41.03% vs. 15.57%, *P* < 0.0001). Moreover, testosterone use and corticosteroid use were significantly more prevalent in the ED cohort compared to the non-ED cohort (2.72% vs. 0.21%, *P* < 0.0001 and 11.45% vs. 6.05%, *P* < 0.0001, respectively). Patients in the ED cohort also had significantly more wrist fractures compared to those in the non-ED cohort (2.67% vs. 2.08%, respectively, *P* = 0.0138), vertebral fractures (4.38% vs. 2.59%, respectively, *P* < 0.0001), and rib fractures (4.36% vs. 3.05%, respectively, *P* < 0.0001). Of the 4636 patients in the ED cohort, 59 (1.27%) developed hip fracture during a median observation time of 5.4 years (interquartile range = 2.9–7.6 years). The incidence of hip fracture was significantly higher (*P* < 0.0001) in the ED cohort compared to the non-ED cohort: only140 of the 18,544 (0.75%) age-matched controls in the non-ED cohort developed hip fracture during a median observation time of 10.8 years (interquartile range = 7.9–13.5 years). During follow-up, the period during which hip fracture developed was significantly shorter in the ED cohort (5.4 years) compared to the non-ED cohort (10.8 years).

**Table 1:** Baseline characteristics of patients with and without erectile dysfunction in Taiwan, 1996–2010, n = 23,180.

Variables	Erectile dysfunction		P value
	Yes	No	
	N = 4,636	N = 18,544	
**Hip fracture patients, n (%)**	59(1.27)	140 (0.75)	0.0006
**Period of developing hip fracture median (IQR), years**	5.4(2.9–7.6)	10.8(7.9–13.5)	<0.0001
**Age mean (SD), years**	58.0(10.4)	58.0(10.8)	0.7481
**Age group, n (%)**			
40–49	1198(25.84)	4792(25.84)	
50–59	1561(33.67)	6244(33.67)	
60–69	1147(24.74)	4588(24.74)	
70–79	655(14.13)	2620(14.13)	
≥80	75(1.62)	300(1.62)	1.0000
**Charlson Comorbidity Index, n (%)**			
0	246(5.31)	4529(24.42)	
1–2	1177(25.39)	7196(38.81)	
3–4	1311(28.28)	3931(21.20)	
≥5	1902(41.03)	2888(15.57)	<0.0001
**Co-morbidity, n (%)**			
Hypertension	3132(67.56)	8273(44.61)	<0.0001
Diabetes mellitus	1923(41.48)	4037(21.77)	<0.0001
Hyperlipidemia	2872(61.95)	6914(37.28)	<0.0001
Osteoporosis	468(10.09)	680(3.67)	<0.0001
Chronic kidney disease	1069(23.06)	1918(10.34)	<0.0001
Chronic liver disease	2543(54.85)	6492(35.01)	<0.0001
Chronic pulmonary disease	2690(58.02)	6836(36.86)	<0.0001
Hyperthyroidism	239(5.16)	429(2.31)	<0.0001
Hyperparathyroidism	5(0.11)	33(0.18)	0.2913
Dementia	333(7.18)	414(2.23)	<0.0001
Stroke	681(14.69)	960(5.18)	<0.0001
Epilepsy	150(3.24)	351(1.89)	<0.0001
Depression	953(20.56)	1441(7.77)	<0.0001
Parkinson’s disease	195(4.21)	280(1.51)	<0.0001
**Medication, n (%)**			
Testosterone	126(2.72)	39(0.21)	<0.0001
Corticosteroids	531(11.45)	1122(6.05)	<0.0001
**Relevant fracture, n (%)**			
Wrist fracture	124(2.67)	386(2.08)	0.0138
Vertebral fracture	203(4.38)	480(2.59)	<0.0001
Rib fracture	202(4.36)	565(3.05)	<0.0001

IQR: interquartile range; SD: standard deviation.

### Incidence rate and risk of hip fracture

Table 2 stratifies the incidence densities and HRs for hip fracture in the ED and non-ED cohorts during the follow-up period. During follow up, 59 (1.27%) patients in the ED cohort and 140 (0.75%) patients in the non-ED cohort developed hip fractures. The overall incidence of hip fracture was 3.74-times higher in the ED cohort than in the non-ED cohort (2.03 vs. 0.50 per 1000 person-years, respectively) after adjusting for age, CCI, and related comorbidities (hypertension, diabetes mellitus, hyperlipidaemia, osteoporosis, chronic kidney disease, chronic liver disease, chronic pulmonary disease, hyperthyroidism, hyperparathyroidism, dementia, stroke, epilepsy, depression, Parkinson’s disease, and use of testosterone or corticosteroids). Compared to the non-ED cohort, the ED cohort consistently had a significantly higher incidence of hip fracture in all age groups, and the incidence rate increased with age. Additionally, hip fracture risk in the ED cohort was significantly higher in younger patients compared to older patients (HR = 4.89, 95% CI = 2.55–9.37, *P* < 0.0001). In both the ED and non-ED cohorts, the follow-up duration was significantly associated with the hip fracture incidence rate. However, incidence rate of hip fractures within 1 year of ED diagnosis was higher in the ED cohort than in the non-ED cohort (0.68 vs. 0.11 per 1000 person-years, respectively). The difference between the two groups was largest at the 3-year follow up (HR = 7.85, 95% CI = 2.94–20.96, *P* < 0.0001).

**Table 2:** Incidence of hip fracture at different age stratification and different follow-up duration among patients with or without erectile dysfunction.

Variables	Patients with erectile dysfunction			Patients without erectile dysfunction				
	Hip fracture	PYs	Rate	Hip fracture	PYs	Rate	IRR (95% CI)	Adjusted HR^a^ (95% CI)
**Overall**	59	29097.97	2.03	140	277495.91	0.50	4.02 (2.96–5.45)^b^	3.74 (2.55–5.48)^b^
**Stratify age**								
40–59	15	16531.20	0.91	38	165336.80	0.23	3.95 (2.17–7.18)^b^	4.89 (2.55–9.37)^b^
≥60	44	12566.77	3.50	102	112159.11	0.91	3.85 (2.70–5.48)^b^	3.42 (2.24–5.22)^b^
**Follow-up time, year**								
1	3	4433.06	0.68	2	18543.31	0.11	4.02 (2.96–5.45)^b^	4.70 (0.68–32.33)^c^
3	17	12103.71	1.40	7	55624.41	0.13	11.16 (4.63–26.91)^b^	7.85 (2.94–20.96)^b^
5	27	18426.91	1.47	12	92692.63	0.13	11.32 (5.73–22.34)^b^	6.91 (3.24–14.71)^b^

PYs, person-years; Rate, incidence rate in per 1000 person-years; IRR, incidence rate ratio; 95% CI, 95% confidence interval; HR, hazard ratio;Follow-up time, the follow-up time after the index date of erectile dysfunction diagnosis.

^a^Model adjusted for age, Charlson Comorbidity Index and related comorbidities (hypertension, diabetes mellitus, hyperlipidaemia, osteoporosis, chronic kidney disease, chronic liver disease, chronic pulmonary disease, hyperthyroidism, hyperparathyroidism, dementia, stroke, epilepsy, depression, Parkinson’s disease, and use of testosterone or corticosteroids).

^b^P<0.0001;

^c^ P = 0.1157.

**Fig 2:**
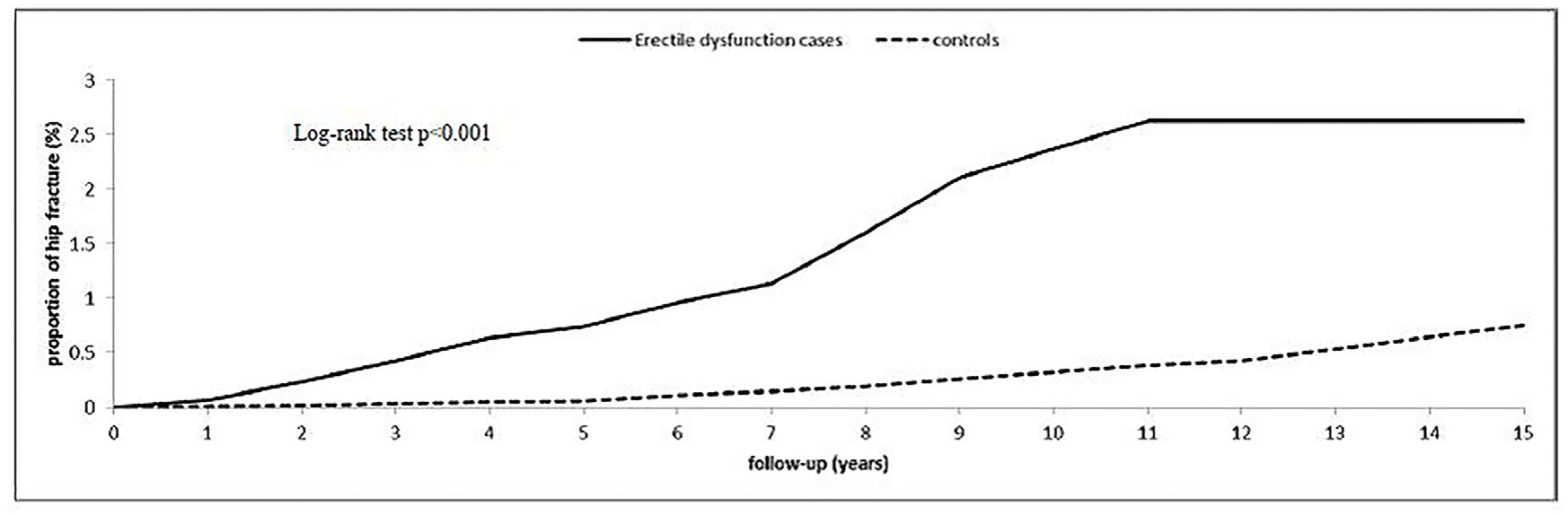
Cumulative incidence of hip fracture for adult patients with erectile dysfunction and the general population control cohort.

Fig 2 presents the Kaplan–Meier curves for the cumulative incidences of hip fracture after 15 years of follow up in the ED and non-ED cohorts. The 1-,5-,10-, and 15-year actuarial rates of hip fracture incidence were 0.068%, 0.741%, 2.370%, and 2.630% in the ED cohort, respectively, and 0.011%, 0.065%, 0.324%, and 0.755% in the non-ED cohort, respectively.

Table 3 summarizes the ED types and the corresponding relative risks and HR for the incidence of hip fracture. Compared to patients in the non-ED cohort, patients with psychogenic ED in the ED cohort had a 1.27-fold higher risk of developing hip fracture, which was not statistically significant (95% CI = 0.30–5.28; *P* = 0.7465). However, patients with organic ED had a 4.01-fold higher risk of developing hip fractures, which was statistically significant (95% CI = 2.73–5.90; *P* < 0.0001).

**Table 3:** Incidence rates and hazard ratios of hip fracture risk in patients with different types of erectile dysfunction.

Variables	N	Hip fracture	PYs	Rate	IRR (95% CI)	Adjusted HR^a^ (95% CI)
**Without erectile dysfunction**	18544	140	277495.91	0.50	ref	ref
**With erectile dysfunction**	4636	59	29097.97	2.03	4.02 (2.96–5.45)^c^	3.74 (2.55–5.48)^c^
**Psychogenic erectile dysfunction**	208	2	1835.56	1.09	2.16 (0.53–8.72)^b^	1.27 (0.30–5.28)^d^
**Organic erectile dysfunction**	4428	57	27262.41	2.09	4.14 (3.05–5.64)^c^	4.01 (2.73–5.90)^c^

PYs, person-years; Rate, incidence rate in per 1000 person-years; IRR, incidence rate ratio; 95% CI, 95% confidence interval; HR, hazard ratio;

^a^Model adjusted for age, Charlson Comorbidity Index and related comorbidities (hypertension, diabetes mellitus, hyperlipidaemia, osteoporosis, chronic kidney disease, chronic liver disease, chronic pulmonary disease, hyperthyroidism, hyperparathyroidism, dementia, stroke, epilepsy, depression, Parkinson’s disease, and use of testosterone or corticosteroids).

^b^ P = 0.2796;

^c^ P<0.0001;

^d^ P = 0.7465.

Table 4 presents the results of the Cox regression analysis and the three risk factors for hip fracture in the ED cohort: age, osteoporosis, and Parkinson’s disease. Of all comorbidities, osteoporosis was the largest risk factor for hip fracture. Therefore, ED and osteoporosis were evaluated for associations with hip fracture risk. Table 5 summarises the results.

**Table 4:** Cox regression model: significant predictors of hip fracture after erectile dysfunction (n = 4,636).

Variables	Adjusted HR ^a^	(95% CI)	P-value
**Age (in 10-year interval)**	1.45	(1.11–1.90)	0.0070
**Osteoporosis**	5.44	(3.14–9.43)	<0.0001
**Parkinson’s disease**	2.50	(1.30–4.82)	0.0061

HR, hazard ratio; 95% CI, 95% confidence interval.

^a^Model adjusted for age, Charlson Comorbidity Index and related comorbidities (hypertension, diabetes mellitus, hyperlipidaemia, osteoporosis, chronic kidney disease, chronic liver disease, chronic pulmonary disease, hyperthyroidism, hyperparathyroidism, dementia, stroke, epilepsy, depression, Parkinson’s disease, and use of testosterone or corticosteroids).

**Table 5:** Cox proportional hazard regression analysis for interaction of osteoporosis and erectile dysfunction on the risk of hip fracture.

Variables		N	Hip fracture	Adjusted HR(95% CI)	Adjusted HR(95% CI)	*P*-value^a^
Erectile dysfunction	Osteoporosis					
No	No	17864	108	ref		<0.0001
No	Yes	680	32	4.09 (2.67–6.24)^b^		
Yes	No	4168	28	3.67 (2.29–5.88)^b^	ref	
Yes	Yes	468	31	15.66 (9.59–25.58)^b^	4.27 (2.50–7.28)^b^	

HR, hazard ratio; 95% CI, 95% confidence interval.

^a^*P*-value for interaction;

^b^ P<0.0001.

Table 5 summarises the joint effects of ED and osteoporosis on hip fracture risk. Compared to non-ED patients without osteoporosis, hip fracture risk was higher in non-ED patients with osteoporosis (HR = 4.09, 95% CI = 2.67–6.24), in ED patients without osteoporosis (HR = 3.67, 95% CI = 2.29–5.88), and in ED patients with osteoporosis (HR = 15.66, 95% CI = 9.59–25.58). In the ED cohort, hip fracture risk was significantly higher in patients with osteoporosis (HR = 4.27, 95% CI = 2.50–7.28) than in patients without osteoporosis. Additionally, ED and osteoporosis had a significant interaction (interaction, P < 0.0001)

## Discussion

The link between ED and hip fracture has not been comprehensively studied. To our knowledge, this is the first nationwide population-based study to investigate the relationship between ED and subsequent hip fracture in an Asian population. During the follow-up period, 59 (1.27%) patients in the ED cohort and 140 (0.75%) patients in the non-ED cohort developed hip fracture. After adjusting for potential confounding factors, the ED cohort had a 3.74-fold higher risk of hip fracture compared to the non-ED cohort. Organic ED was associated with a relatively higher hip fracture risk and HR. In the ED cohort, hip fracture risk was particularly high in those aged 40–59 years. Additionally, the joint effect of ED and osteoporosis on hip fracture risk was revealed. The results of this population-based cohort study revealed a significant association between ED and hip fracture risk. Similar results were obtained in a prospective population-based study of fractures in 4,696 elderly Danish men by Frost. M et al. [17], which first revealed a higher osteoporotic fracture risk in men with self-reported with ED compared to controls (HR = 2.04; 95%CI 1.31–3.18). However, the 4,696 participants were slightly older than our study population and were not compared to the age-matched background population. Furthermore, ED was evaluated only by asking, “Do you suffer from impotence?”. The possible answers were categorical (yes/no) and were not confirmed by a specialist. Additionally, the data were subject to recall bias and information bias. Nevertheless, Frost. M et al [17] is the first report of an association between ED and fracture risk. However, the exact aetiology and pathogenesis of ED-related hip fractures need further study.

The two major ED subtypes are lifelong ED, in which erection cannot be achieved after the onset of sexual desire, and acquired ED, in which ED begins after a period of normal sexual and erectile activity. Each subtype can have either psychogenic or organic contributors. ED can generally be classified as organic, psychogenic, or mixed, depending on the pathogenic mechanism. In organic ED, the contributing factors may include structural disorders or neurogenic, vasculogenic, endocrinologic, medical or urologic disorders [18,19]. Organic ED can be excluded if the patient has morning or situational erections [20]. In psychogenic ED, the predominant or exclusive cause of ED is a positive psychosocial factor such as depression, performance anxiety or relationship problems [19–21]. Psychogenic ED is the correct diagnosis if no organic cause of ED is detected [22]. In mixed ED, the contributing factors are a combination of organic and psychogenic factors. Notably, recent studies suggest that ED is usually mixed, i.e., the patient exhibits both organic and psychogenic factors [19,23]. However, since the ICD-9-CM does not include a diagnostic code for mixed ED, patients with mixed ED are conventionally coded as organic ED [24].

ED often occurs in association with other disorders such as diabetes, cardiovascular disease (CVD), hypertension, obesity, Parkinson’s disease, depression, and chronic obstructive pulmonary disease (COPD) [25–27]. Based on the literature on risk factors for falls in older people, we hypothesized that several chronic conditions increase the risk of hip fracture. One risk factor is hormonal imbalance. Testosterone plays a critical role in the pathogenesis of fall-related hip fractures through its effect on muscle strength. Patients with ED exhibit androgen deficiencies that can directly or indirectly induce depression, dementia, sleep disorders, and osteoporosis, all of which can eventually contribute to hip fracture. Osteoporosis in particular increases the risk of hip fracture from a fall because it reduces bone strength [28–32]. Several plausible mechanisms of the increased risk of hip fracture in patients with ED have been proposed. One proposed mechanism is depression. In a longitudinal study of Asian men with ED, Chou et al. reported a high risk of developing depression, particularly within 1 year of ED diagnosis. Chou et al. also reported that patients with ED had a 2.24-fold higher HR for developing depression compared to controls without ED [31]. Diem et al. reported that depression symptoms in elderly males are associated with an approximately 1.8-fold increase in the annualised rate of bone loss in the hip, femoral neck, and trochanter bones [33]. Their findings are consistent with previous cross-sectional studies that have reported a lower bone mineral density (BMD) in patients with depression than in patients without depression [34–37]. A meta-analysis of patients with a history of depression further revealed a high risk of dementia later in life [38]. Since cognitive dysfunction, including dementia and depression, is a major risk factor for falls, we infer that a poor mental status caused by ED may be a risk factor for the development of hip fracture. Third, in a cross-sectional study by Moore et al., a cognitive comparison between men with and without ED revealed poorer cognitive performance in men with ED. The authors concluded that ED may early indicate a compromised cognition and predict further cognitive health problems [39,40]. A 7-year follow-up study by Yang et al. investigated the risk of dementia in an Asian population of patients with ED. The authors reported that, in patients with ED, the incidence rate of dementia was 35.33 per 10,000 person-years, which was1.68-times higher than that in the comparison cohorts without ED [40]. Cognitive impairments markedly increase the risk of falls resulting in hip fractures [41,42]. Fourth, vitamin D deficiency is a major risk for hip fracture in people in institutions [43]. In a study of 143 cases of ED, Barassi et al. reported a higher vitamin D deficiency in patients with arteriogenic ED than in patients with non-arteriogenic ED. The authors also reported that patients with severe ED had low serum vitamin D levels. Notably, however, the patients analyzed in their study were asymptomatic, and no patients reported using vitamin D supplements [7]. Thus, vitamin D deficiency is a possible cause of the increased hip fracture risk in patients with ED. Furthermore, in a large-scale cohort study comprising 32,616 US men, Gao et al. reported that compared to healthy controls without ED, men with ED were 3.8-times more likely to develop Parkinson’s disease, which is a risk factor for falls [44]. Finally, studies suggest that CVD is the most common comorbid condition in men with ED. For example, in Vlachopoulos et al., a review of data for 92,757 participants in 14 studies showed that, compared to patients without ED, patients with ED had a 44% higher risk of CVD-related events, a 62% higher risk of MI, and a 25% higher overall mortality risk [45]. A literature review by Gandaglia et al. concluded that ED and CVD should be considered separate manifestations of the same systemic disorder. The link between these two conditions lies in the interaction between CVD risk factors, androgens, and chronic inflammation leading to atherosclerosis and flow-limiting stenosis. ED usually precedes an onset of CVD and can be considered an early marker of symptomatic CVD [46]. Studies of populations in both Taiwan and Sweden have reported an association between CVD and the risk of major osteoporotic fracture [47–49]. Lai et al. demonstrated that the overall fracture risk was approximately 1.2-fold higher in a CVD cohort than in a non-CVD cohort [47]. Finally, in Koseoglu et al., a prospective study of 60 males with COPD revealed ED in over 75% of the patients [50]. In a retrospective cohort study by Shen et al., the overall incidence rate of ED was 1.88-fold higher in the COPD cohort than in the non-COPD cohort (24.9 vs. 13.3 per 1000 person-years, respectively; 95% CI = 1.61–2.18) [51]. Because COPD is associated with loss of muscle strength and is a risk factor for osteoporosis [52], these findings further support the correlation between ED and hip fracture.

A major strength of the present study is the use of population-based data that are highly representative of the general population of Taiwan. Although this cohort study identified an association between ED and hip fracture risk, several limitations of this study must be considered. First, diagnoses of hip fracture and ED were identified by ICD-9-CM codes in the database, but no information regarding the accuracy of these codes is available. This study was performed using data in the NHIRD, in which all clinically diagnoses are made by the physician in charge. Moreover, the International Index of Erectile function (IIEF-5) score for identifying ED-patients were not recorded. Besides, the identification of ED subtypes was depended in part on the physician’s judgment, which means inevitable subjectivity. However, this subjective bias could be reduced by medical experts of the BNHI Professional Peer Review Committee by whom conduct regular check up to ascertain the accuracy of diagnostic codes used in health insurance claims in Taiwan. Doctors and hospitals who did not diagnose according to standard clinical criteria and guidelines and enter diagnostic codes accurately would be subject to big fines.

Second, private information such as disease severity, body weight, imaging results, tobacco use, exercise habit, Androgen Deficiency in the Aging Male (ADAM) scores, biochemistry profiles, and nutritional status, were unavailable. Another issue is that an underestimated incidence of ED is expected because of the embarrassing nature of this condition. Men with erectile problems may prefer to seek private treatment or buy drugs over the Internet. Finally, the statistically significant associations identified in this study may not be clinically significant associations. Without access to detailed individual medical records, a population-based study cannot ascertain the exact pathology of the association between ED and hip fracture.

## Conclusion

In conclusion, this study successfully identified an association between ED and hip fracture. Compared to the general population, men with ED are at a significantly higher risk of developing hip fracture. That is, ED can be considered an early predictor of hip fracture. Patients with ED, particularly those aged 40–59 years, should receive early examinations for BMD.
